# Cervical dysplasia and cancer and the use of hormonal contraceptives in Jamaican women

**DOI:** 10.1186/1472-6874-8-9

**Published:** 2008-05-30

**Authors:** Norma McFarlane-Anderson, Patience E Bazuaye, Maria D Jackson, Monica Smikle, Horace M Fletcher

**Affiliations:** 1 Department of Basic Medical Sciences, University of the West Indies, Mona Campus, Kingston, Jamaica; 2 Department of Community Health & Psychiatry, University of the West Indies, Mona Campus, Kingston, Jamaica; 3 Department of Microbiology, University of the West Indies, Mona Campus, Kingston, Jamaica; 4 Department of Obstetrics & Gynaecology. University of the West Indies, Mona Campus, Kingston, Jamaica

## Abstract

**Background:**

This study was conducted to determine whether use of hormonal contraceptives is associated with cervical dysplasia and cancer in a population where there is widespread use of hormonal contraception and the rates of cervical cancer remain high at 27.5/100,000.

**Methods:**

A case-control study was conducted among women visiting the colposcopy and gynaelogical clinics at a tertiary referral hospital. Two hundred and thirty six cases CIN I (72), II (59), III (54), cancer (51) and 102 controls, consented and were interviewed on use of contraceptives using a structured questionnaire. Logistic regression was used to determine odds ratios (ORs) and 95% confidence intervals (CIs) associated with use of hormonal contraception in cases and controls and in low and high risk cases. Recruitment was carried out from 2001–2002.

**Results:**

Contraceptives used were: oral contraceptives – 35%, injections (depot medroxy progesterone acetate (Depo-provera) – 10%, Intrauterine devices – 2%, combinations of these and tubal ligation – 30%. 23% reported use of 'other' methods, barrier contraceptives or no form of contraception. Barrier contraceptive use was not significantly different between cases and controls. Current and/or past exposure to hormonal contraceptives (HC) by use of the pill or injection, alone or in combination with other methods was significantly higher in the cases. In multivariate analysis with age and number of sexual partners as co-variates, use of hormonal contraception was associated both with disease, [OR, 1.92 (CI 1.11, 3.34; p = 0.02] and severity of the disease [OR, 2.22 (CI 1.05, 4.66) p = 0.036]. When parity and alcohol consumption were added to the model, hormonal contraception was no longer significant. The significant association with high risk disease was retained when the model was controlled for age and number of sexual partners. Depo-provera use (with age and number of sexual partners as covariates) was also associated with disease [OR, 2.43 (CI 1.39, 4.57), p = 0.006] and severity of disease [OR 2.51 (1.11, 5.64) p = 0.027]. With parity and alcohol added to this model, depo-provera use retained significance. Exposure to HC > 4 years conferred more risk for disease and severity of disease.

**Conclusion:**

Hormonal contraception did confer some risk of dysplasia and women using HC should therefore be encouraged to do regular Pap smear screening.

## Background

The search for links between the use of hormonal contraception or hormone replacement therapy and the development of some reproductive system cancers in women has yielded conflicting results. Thus there is evidence of a correlation between estrogen and increased risk of breast cancer while on the other hand, it has been suggested that use of oral contraceptives for one year or more is protective against endometrial and ovarian cancers with the protective effect lasting for at least 10 years [1]. There are several studies which have reported that hormonal contraception (HC) – pills and injectables – moderately increase the risk of cervical cancer as well as being a risk for all stages of cervical cancer [2-8] particularly in human papilloma virus (HPV)-positive women thus suggesting that oral contraceptives may act as a promoter for HPV-induced carcinogenesis [4,5].

The inconsistent reports of an association between hormonal contraception and cervical dysplasia and cancer may be related, in part, to confounding risk factors that include sexual and lifestyle behaviours [2,3]. The causal link between HPV and cervical dysplasia and cancer is now generally accepted [9-13]. In addition to any direct effect of HC on the development of cervical dysplasia, either as an initiator or promoter of carcinogenesis [4,5], the use of hormonal contraceptives could result in women indulging in more unprotected sexual activity putting them at more risk of HPV and other STI infections and their sequelae.

Since the 1960s, use of HC for family planning has been actively encouraged in Jamaica. In addition to pills, Depot medroxy progesterone acetate (DMPA) injections and lovonorgestrel implants are available. Data from a Reproductive Health Survey by the National Family Planning Board (2002) showed that in addition to a doubling in the use of condoms, since 1989, there has been a 48% increase in the use of injectables (22.7% vs 33.6%) in women reporting ever use of contraception. Over the period, pill use rose from 47.6% to 56.5% [14].

Given the high incidence of cervical dysplasia [15] and that cervical cancer rates remain high at 27.5/100,000 [16], we looked at whether HC use in the population was a factor in the development of cervical dysplasia and cancer.

## Methods

### Study design and subjects

Ethical approval was obtained from the University Hospital of the West Indies (UHWI) Ethical Review board. Both cases and the comparison group were recruited at the UHWI between April 2001–August 2002. Cases were enrolled from twice weekly conducted colposcopy clinics while women in the comparison group were recruited from weekly gynaecology clinics at the same hospital. Both clinics receive referrals from primary care clinics, hospitals and private practitioners. Consecutive women were invited to participate in the study.

#### Cases

Women with abnormal Pap smears who had been referred to the Colposcopy clinic and had a diagnosis that was confirmed by colposcopic biopsy histology served as the sampling frame. Cases were identified from the clinic registry where they were documented according to the degree of cervical intraepithelial neoplasia (CIN) as CIN-I, CIN-II, CIN-III and cervical cancer.

#### Comparison group

Women attending routine gynaecological clinics who had normal Pap smears were regarded to be disease-free and served as the comparison group.

The study population consisted of CIN-I (n = 72), CIN-II (n = 59), CIN-III (n = 54), cervical cancer (n = 51) and controls (n = 102). When grouped according to severity of disease using the Bethesda classification, there were 72 with low grade lesions (LGSIL) and 164 with high grade lesions (HGSIL). Informed consent was sought after an explanation of the study and those who agreed were asked to sign a consent form. Confidentiality and anonymity were assured before they were interviewed using a structured questionnaire by a single female interviewer. Data on drinking, smoking and other behavioural variables and sexual characteristics were collected. Participation rates among cases and the comparison group were 72% and 60% respectively.

#### Exclusion criteria

The following persons were excluded from the investigation: women who were pregnant, had hysterectomies or were previously diagnosed with adenocarcinoma.

### Statistical Analyses

Differences between cases and the comparison group were examined by the t-test and χ^2 ^as appropriate. Logistic regression was used to determine the association of reproductive and lifestyle factors with disease (presence and severity). Odds Ratios (OR) and confidence intervals (CI) are presented. Analyses were performed using the SPSS software (version 12.0). Statistical significance was achieved when p < 0.05.

## Results

Table 1 shows the characteristics of women with cervical dysplasia and the comparison group and their pattern of contraceptive use. They were of similar age, had about the same number of sex partners but the cases had had more children, had used Pap smear screening more infrequently and were more likely to have consumed alcohol. The methods of contraception used by women in this study were: oral contraceptives (OC) – 35%, DMPA injections – 10%, Intrauterine devices (IUD) – 2%, combinations of OC, DMPA, IUD, and tubal ligation – 30%. The remaining 23% reported use of 'other' methods, barrier contraceptives or no form of contraception. The majority of the women did not use barrier contraceptives; cases 80.5% and comparison group 68.8%. Non – use of barrier contraceptives was not associated with development of disease [OR, 0.63 (CI 0.37, 1.08; p = 0.094].

**Table 1:** Characteristics of women with cervical dysplasia (n = 240) and the comparison group (n = 102). and their patterns of contraceptive use.

***Variable***	**Cases**	**Comparison group**
Age (years, mean ± SD)	39.1 ± 11.8	38.1 ± 10.0
**Reproductive & lifestyle factors:**		
Number of lifetime sex partners (mean ± SD)	4.9 ± 2.1	4.6 ± 2.9
Parity (mean ± SD)	2.8 ± 2.1	2.1 ± 2.4
Regular use of pap smear (%)	32.3	47.7
Consuming alcohol (%)	21.1	10.4
***Contraceptive use % (n)*:^a^**		
None	16.6 (40)	28.8 (29)
Pills	30.4 (73)	34.3 (35)
Injections	11.3 (27)	5.8 (6)
Intra Uterine Device (IUD)	1.7 (4)	2.0 (2)
Combinations – Pills, Injections, IUD	26.3 (63)	13.7 (14)
Combinations – Pills, Injections, tubal ligation	4.2 (10)	7.8 (8)
Other	0.4 (1)	1.0 (1)

^a ^22 cases and 7 subjects from the comparison group missing.

Current and/or past exposure to hormonal contraceptives by use of the pill or injection, alone or in combination with other methods was significantly higher in the cases (79.4%) compared to the comparison group (67.4%) p = 0.023. There was also a significant difference between women classified as high risk (HGSIL) (82.9%) and low risk LGSIL (69.8%) (Bethesda classification) p = 0.032 (Table 2). When women who had used DMPA were compared to women who had never used this method, there were also significant differences in presence of and severity of disease (p = 0.003 and 0.012) respectively.

**Table 2:** Percentage of women exposed to hormonal contraceptives (HC) and depot medroxy progesterone acetate (DMPA) by presence and severity of disease

	**Presence of disease**			**Severity of disease**		
**Contraceptive used**	Comparison					
	Cases	Group	p value	HGSIL^a^	LGSIL^b^	p value

HC^c^	79.4	67.4	0.023	82.9	69.8	0.032
DMPA^d^	69.0	46.7	0.003	75.0	53.7	0.012

^a ^LGSIL – low risk lesions

^b ^HGSIL – high risk lesions

^c ^HC = ever use of hormonal contraceptives by use of the pill, injection or in combination with other methods.

^d ^DMPA = women who reported ever use of injections alone or in combination with other methods.

With age and number of sexual partners as co-variates, use of HC was associated both with presence of disease [OR 1.92 (CI 1.11, 3.34); p = 0.02] and the severity of the disease, [OR 2.22 (CI 1.05, 4.66); p = 0.036 ]. When parity and alcohol consumption (predictors of disease), were added to the model, HC use was no longer significant [OR, 1.59, (CI 0.87, 2.82); p 0.13]. Significant associations were observed for high risk disease when HC, age and number of sexual partners (predictor for severity of disease) were included in the model, [OR = 2.22, (CI 1.05, 4.66) p = 0.036] (Table 3). When use of barrier contraceptives was added to the model, the association of disease and the severity of disease with use of HC was virtually unchanged, [OR,1.95 (CI 1.13, 3.38); p = 0.017] and [OR, 2.21 (CI 1.08, 4.53); p = 0.031], respectively.

**Table 3:** Odds Ratios and Confidence Intervals for hormonal contraceptive effect (HC), medroxy progesterone acetate (DMPA) effect, >4 year HC use on (a). presence and (b). severity of disease.

	**Presence of disease**	**Severity of disease**	
Model	**OR (CI)**	**OR**	**CI**
**Hormonal contraceptive use:**			
Never used	1.0 (reference)	1.0 (reference)	
HC^a^	1.92 (1.11, 3.34)	2.22 (1.05, 4.66)	
HC + parity + alcohol intake ^ab^	1.59 (0.87, 2.82)		
**DMPA use:**			
Never used	1.0 (reference)	1.0 (reference)	
DMPA^a^	2.43 (1.39, 4.57)	2.51 (1.11, 5.64)	
DMPA + parity + alcohol intake^ab^	2.48 (1.30, 4.74)		
**Length of exposure:^a^**			
Never used	1.0 (reference)	1.0 (reference)	
1–4 years	1.47 (0.80, 2.71)	1.68 (0.73, 3.89)	
>4 years HC use	3.56 (1.81, 7.00)	4.43 (1.84, 10.67)	

^a ^Models were adjusted for age and number of sexual partners.

^ab ^Variables entered; age and number of sex partners, variables offered: parity and alcohol consumption.

Of the 236 women who had used HC, 128 (57.2%) reported ever use of DMPA. When these women were compared to those who had never used HC, the percentage of cases who were ever users of DMPA was significantly greater than the controls (χ^2 ^= 8.99, p = 0.03). There were also more DMPA users among the HSIL compared to the LSIL (χ^2 ^= 6.26, p = 0.012). The ORs also shown in Table 3, were slightly higher than those for use of HC, e.g. 2.43 vs 1.92 and 2.51 vs 2.22. DMPA use did retain significance when parity and use of alcohol were included in the model. The effect of DMPA on severity of disease was retained when lifetime sexual partners were added to the model.

Length of exposure to HC varied from one week to greater than ten years with 47.5% having used HC for >4 years. Of the controls, 18.2% and 8.3% had been exposed to HC for <4 years and >4 years respectively while among the cases, percentages exposed were 34.3% and 39.3% respectively. These differences were significant, p = 0.002. Among cases there was also a significant association with high risk disease, p = 0.005. With adjustment for age and number of sexual partners, duration of exposure showed a significant association with disease [OR, 3.56 (CI 1.81, 7.00; p = 0.001)] and severity of disease [OR 4.43 (1.43, 5.02); p = 0.017] (Table 3). There were no data available on length of use of DMPA only.

## Discussion

In this population, current and/or past exposure to HC by use of the pill or injection, alone or in combination with other methods, was significantly higher in cases. Analysis indicates an independent role for HC use especially as it relates to the severity of disease whilst presence of the disease as a result of HC use was modified by the factors that were found to predict the disease. The majority of the women, cases and controls, did not use barrier contraceptives and so were at risk of multiple infections, inclusive of HPV. The duration of the exposure was important such that women who had been using HC for more than four years were at greater risk for both disease and severity of disease. The authors of an earlier study of carcinoma-in -situ (CIS) patients suggested that risk of CIS may be confined to long term users [17].

Several studies have contributed to the present understanding of the relationship between the use of HC and disease. The International Agency for Research on Cancer (IARC) pooled data from eight studies of invasive cancer and two of carcinoma in situ in HPV positive cases and found that risk increased significantly with parity [18]. Women who used HC for less than 5 years were not at increased risk but risk became apparent after 10 years [18]. Results from the WHO collaborative Study of Neoplasia and Steroid Contraceptives showed that there was a slight risk for ever-users that increased with duration of > 4 years [7]. Similar findings were reported by the Oxford Family Planning Association contraceptive study [19,20]. Based on the most recent evaluation of several studies, the IARC has concluded that HC can be classified as carcinogenic to the cervix as well as to the breast[21]. Given that HPV infection is now considered the major factor in the development of cervical cancer, it would seem that HC may be acting as an enhancer of neoplastic growth. Since estrogen is known to have trophic effects consistent exposure to above normal levels could result in this growth. Some investigators found that women with higher levels of estrogen receptor transcripts were significantly more likely to have cervical HPV infection and that estrogen binds to specific DNA sequences within transcriptional regulatory regions on the HPV DNA[22]. It has also been shown that HPV-18 E6 and E7 proteins directly interacted with the estrogen receptor[23]. Cervical ectopy, common in women using HC, [24] could also increase susceptibility to HPV infection. The fact that the association with HC and DMPA exposure is modified by lifestyle covariates suggests that HC, usually associated with less use of barrier protection during sexual contact (exposure to other infections), as well as direct effects of the HC could explain the associations. We have found that bacterial vaginosis is a risk factor for HPV infection (unpublished observations).

Whereas we did not have data on HPV infection in the comparison group in this study, our findings of high rates of HPV infection among healthy pregnant and non pregnant women in Jamaica [25], suggests that the controls in this study were very likely to be infected with HPV.

The data indicates that fewer than 50% of the women underwent regular screening by Pap smear, while in a smaller study of university students only 27% reported screening [26]. Since hormonal contraception is widely used and its use in our population is in fact encouraged, it is imperative that such women be educated and encouraged to do regular Pap smears to enhance early detection.

## Conclusion

Use of HC confers some risk of dysplasia which may be modified/confounded by use of barrier contraceptives, increased number of sexual partners, increased number of biological fathers, parity and alcohol consumption. Given the wide use of HC, regular Pap smear screening should be encouraged.

## Competing interests

The authors declare that they have no competing interests.

## Authors' contributions

NA conceived of the study, carried out statistical analyses and drafted the manuscript. PB was responsible for recruitment and data collection. MJ participated in design and statistical analyses. MS and HF advised on design and recruitment. All authors contributed to the writing of the manuscript and read and approved the final manuscript.

## Pre-publication history

The pre-publication history for this paper can be accessed here:


